# Modeling lifetime greenhouse gas emissions associated with materials for various end-of-life treatments

**DOI:** 10.1007/s10661-018-7050-3

**Published:** 2018-11-14

**Authors:** Ian J MacDonald, Satish B. Mohan

**Affiliations:** 1 ReUse Action Inc., 980 Northampton St., Buffalo, NY 14211 USA; 2 Department of Chemistry, Department of Physics. Erie County Community College, State University of New York, 6205 Main Street, Williamsville, NY 14221 USA; 3 State University of New York at Buffalo, 223 Ketter Hall, Buffalo, NY 14260 USA

**Keywords:** Global warming, Global warming potential (GWP), Building materials, Deconstruction, Mathematical modeling, Global Warming Impact of Materials (GWIM)

## Abstract

This research has developed mathematical models for computing lifetime greenhouse gas (GHG) emissions associated with materials. The models include embodied carbon (EC) emissions from the manufacture of materials, and GHG emissions from incineration, or landfill gas (LFG) production from landfill disposal of the material beyond their service lives. The models are applicable to all materials; however, their applications here are demonstrated for the lumber from a residential building with 50- and 100-year service lives, and with incineration, landfill, and deconstruction as end-of-life treatments. This paper introduces a new metric for lifetime GHG emissions associated with materials termed “Global Warming Impact of Materials (GWIM).” The GWIM is subdivided into two portions: (i) productive portion (GWIM_p_) that includes the materials’ emissions until the service life of the facility and (ii) non-productive portion (GWIM_np_) which includes the materials’ GHG emissions beyond the service life until they are eliminated from the atmosphere. In place of the current, static, EC measurements (kgCO_2_e or MTCO_2_e), this model reports the GWIMs in units of kgCO_2_e-years or MTCO_2_e-years, which includes the effects of “time of use” of a facility. Using the models, this paper has computed GHG reductions by deconstruction, with material recoveries of 30%, 50%, and 70% at demolition for reuse, recycle, or repurpose. A 70% material recovery, after a 50-year service life of the building, affected a savings of 47% and 52% if the remaining 30% debris was incinerated or landfilled respectively. All of the values computed using models checked out with manual calculations.

## Introduction

### Greenhouse gases and global warming

Solar energy heats the Earth’s surface where some of it is absorbed and the remaining is radiated back toward space as infrared radiation (IR). For 2000 years, the atmospheric concentrations of GHGs have remained relatively constant, but since the 1700s, the concentration of atmospheric carbon dioxide (CO_2_) has risen about 30%, and the Earth’s average temperature has risen 1.6 ± 0.16 °C (IPCC 2014; Houghton 2005; Keller 2009). Both increases are concomitant and greater than would be expected from natural phenomenon such as volcanism. High atmospheric concentrations of greenhouse gases (GHGs) such as CO_2_ and methane (CH_4_), due to the large-scale burning of fossil fuels such as coal and oil for energy, are known to be responsible for global warming. Some other gases such as nitrous oxide (N_2_O) and refrigerants and dielectric gases are emitted in much smaller quantities than CO_2_, but are important because they absorb IR radiation 25–22,800 times more efficiently and have atmospheric lifetimes of 125–22,880 years (EPA 2017; IPCC 2014). In the USA, CO_2_ accounts for 81% of GHG emissions, while CH_4_, N_2_O, and refrigerants and dielectric gases account for 10.0%, 5.1%, and 2.8% respectively (EPA 2017).

In 2015, the total US GHG emissions and sinks were calculated to be 5827.7 MMTCO_2_e, 17.3% of the global emissions of 33,733 MMTCO_2_e (EPA 2017). Electricity production emitted 1900.7 MMTCO_2_e, and landfills released 115.7 MMTCO_2_e, mainly from CH_4_, produced by anaerobic digestion of organic matter by microbes (EPA 2017). Global warming results from increasing atmospheric GHG concentrations. The ramifications to society are climate change, which is a reorganization of Earth’s weather patterns in response to excess atmospheric energy (Houghton 2005; Held and Soden 2006; Keller 2009; Hansen et al. 2016). Most projections show that climate change will be costly to society, and therefore it is advisable that GHG emissions must be reduced. In the 2015 United Nations global conference on climate change, held in Paris, the USA is committed to reducing its GHG emissions in the range of 17% by 2020, and 26–28% by 2025, relative to 2005 emission levels of 7429 MMTCO_2_e (IPCC 2014; UN 2016). This commitment was later withdrawn in 2017 (Mohan and MacDonald 2016a b).

### Impact of materials

This research has set up mathematical models for computing lifetime GHG emissions associated with materials for service lives of 50 and 100 years, and for three end-of-life treatments of demolition debris: incineration, landfill, and deconstruction. These models are generic and can be applied to any material or assembly of materials; their application in this paper has been demonstrated on lumber from an example residential building.

This paper introduces a new method for measuring lifetime GHG emissions associated with materials termed “Global Warming Impact of Materials (GWIM).” The GWIM is subdivided into two portions: (i) productive portion (GWIM_p_) that includes the materials’ emissions until the service life of the facility and (ii) non-productive portion (GWIM_np_) which includes the materials’ GHG emissions beyond the service life until they are eliminated from the atmosphere. In place of the current, static, EC measurements (kgCO_2_e or MTCO_2_e), this model reports the GWIMs in units of kgCO_2_e-years or MTCO_2_e-years, which includes the effects of “time of use” of a facility.

The research presented in this paper focuses on the global warming impact of materials, specifically from the GHG emissions occurring during their manufacture, and from GHG emissions arising from disposal of construction and demolition (C&D) debris at the end of their service life. Service life, defined as the time of a structure’s productive use, has been included in the models to calculate the productive and unproductive GHG emissions.

Commercial and residential buildings in the USA use 39.4% of all the energy and 67.9% of all the electricity produced, and are responsible for 38.1% of all the CO_2_ produced (AIA 2010). The largest emissions occur during the operation phase of a building’s lifetime where more than 50% of the total CO_2_ emissions occur as a result of heating, cooling, lighting, and powering appliances (DOE 2012). Many agencies are working to improve energy efficiencies for this phase. Generally, the global warming potential (GWP) of building materials is measured by the embodied carbon (EC) of the material. This is the amount of GHG emitted during the manufacture of the material. The EC is a static measure of GWP that does not factor in the length of use of the material or the GHGs emitted after demolition when the C&D debris are disposed of. This work extends EC emissions by including the end-of-life treatments, and models GHG reductions via the use of deconstruction instead of demolition when the building becomes obsolete. This paper has introduced a new concept for measuring the lifetime GHG emissions associated with materials, and termed it Global Warming Impact of Materials (GWIM). For demonstrating the concept, lifetime GHG emissions from building materials have been mathematically modeled for two service lives of 50 years and 100 years, and for three end-of-life treatments at demolition: incineration, landfill disposal, and deconstruction.

Currently, the GHG emissions are measured in kgCO_2_e or MMTCO_2_e. This paper has suggested that GHG emissions be measured in kgCO_2_e-years to include the effect of “time of use,” or service life of a facility. GHG emissions have been computed for two service lives of 50 and 100 years using mathematical models for an example building to demonstrate the effect of service life.

## Materials and methods

### Modeling lifetime GHG emissions or global warming impact of materials

**Fig. 1:**
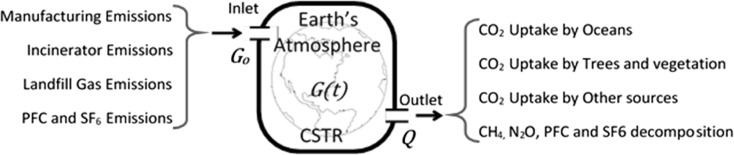
CSTR model of greenhouse gas emissions associated with building materials

In this work, the atmospheric concentrations of GHGs associated with building materials, during manufacturing, and end-of-life treatments were simulated with a continuously stirred tank reactor (CSTR), as is done in chemical engineering. Figure 1 shows the schematic of the CSTR model, where the Earth’s atmosphere is represented by the volume of the CSTR. In this model, *G* is used as a generic variable representing the atmospheric concentration of any GHG. The inlet on the left side of the CSTR represents lifetime GHG emissions into the Earth’s atmosphere. These may come from the manufacture of building materials and from their disposal after the structure is demolished. The GHG emissions associated with manufacturing and from incineration of construction and demolition (C&D) debris are considered instantaneous pulse inputs into the CSTR, as their production times are extremely short. Biogenic carbon is the measure of the amount of GHG emitted when a material is incinerated or used for fuel. In this work, it represents the stored carbon in biological materials such as wood and in organic materials such as plastic. The emission of landfill gas (LFG) from decomposing building materials is a time-dependent source function that gradually releases GHGs into the atmosphere, which depends on the decay rate (*k*) and methane production potential (*L*_o_) of landfilled materials.

The outlet represents the Earth’s physical, chemical, and biological mechanisms that remove CO_2_ and other GHGs from the atmosphere (Trussell and Hand 2005). The removal mechanisms include partitioning of CO_2_ into the oceans, CO_2_ uptake by trees and plants, and oxidation of CH_4_ by OH-radicals in the atmosphere. The removal mechanisms are a function of the atmospheric residence lifetime of the individual GHGs. Residence lifetime is a concept used in chemical engineering that describes the average amount of time a molecule spends in a reactor before exiting (Trussell and Hand 2005). The atmospheric residence lifetimes of CO_2_ and CH_4_ are estimated to be 50–200 years and 12 years respectively (EPA 2017).

The CSTR model is also adaptable to determine the GWP of other GHGs. Refrigerants (e.g., HFCs, CFCs) and dielectric gases (SF_6_) used in electrical distribution equipment also migrate to the stratosphere and are broken down photochemically by sunlight to produce halogen radicals that catalyze the reduction of O_3_ to O_2_.

### GHG concentrations in the atmosphere

The derivation of the equation for the atmospheric concentration of a GHG due to an emission input (manufacturing, landfill disposal, incinerations, fugitive emissions, etc.) is shown below. Symbols for the variables and constants used in the GHG emissions model are defined in Table 1. Equation 1 represents the mass flow of a GHG (*G*) into the atmosphere and a first-order GHG removal mechanism (−*QG*) that is dependent on the atmospheric concentration (*G*) and the rate of removal (*Q*) due to ocean partitioning, plant uptake, etc.

1 $$ {V}_{\mathrm{atm}}\frac{dG}{dt}=- QG $$

**Table 1:** Variables and constants used in modeling GHG emissions

Symbol	Definition	Units
*G*	GHG concentration in the atmosphere	Mass/vol $$ \left(\raisebox{1ex}{$\mathrm{kg}$}\!\left/ \!\raisebox{-1ex}{$\mathrm{L}$}\right.\right) $$
*V*_atm_	Volume of the Earth’s atmosphere	Vol (L)
$$ \frac{dG}{dt} $$	Change in concentration of GHG in Earth’s atmosphere	Mass/vol s $$ \left(\raisebox{1ex}{$\mathrm{kg}$}\!\left/ \!\raisebox{-1ex}{$\mathrm{L}\bullet \mathrm{year}$}\right.\right) $$
$$ \frac{d\left[{\mathrm{CO}}_2\right]}{dt} $$	Change in concentration of carbon dioxide in Earth’s atmosphere	Mass/vol s $$ \left(\raisebox{1ex}{$\mathrm{kg}$}\!\left/ \!\raisebox{-1ex}{$\mathrm{L}\bullet \mathrm{year}$}\right.\right) $$
$$ \frac{d\left[{\mathrm{CH}}_4\right]}{dt} $$	Change in concentration of methane in Earth’s atmosphere	Mass/vol s $$ \left(\raisebox{1ex}{$\mathrm{kg}$}\!\left/ \!\raisebox{-1ex}{$\mathrm{L}\bullet \mathrm{year}$}\right.\right) $$
*Q*	Rate of flow out of the atmosphere	Vol/time $$ \left(\raisebox{1ex}{$V$}\!\left/ \!\raisebox{-1ex}{$\mathrm{year}$}\right.\right) $$
*θ*	Atmospheric residence time of GHG. $$ \theta =\raisebox{1ex}{${V}_{\mathrm{atm}}$}\!\left/ \!\raisebox{-1ex}{$Q$}\right. $$	Time (years)
*θ*_CO2_	Atmospheric residence time of carbon dioxide	Time (years)
*θ*_CH4_	Atmospheric residence time of methane	Time (years)
*t*	Time since the manufacture of a building material	Time (years)
*T*_Demo_	Time of demolition of the building	Time (years)
EC	Embodied carbon. Amount of carbon emitted per mass of building material manufactured	Mass CO_2_/mass material $$ \left(\raisebox{1ex}{$\mathrm{kg}\ {\mathrm{CO}}_2$}\!\left/ \!\raisebox{-1ex}{$\mathrm{kg}$}\right.\right) $$
EC(*t*)	Time-dependent embodied carbon of a building material	Mass CO_2_/mass material $$ \left(\raisebox{1ex}{$\mathrm{kg}\ {\mathrm{CO}}_2$}\!\left/ \!\raisebox{-1ex}{$\mathrm{kg}$}\right.\right) $$
EC_bio_	Embodied biogenic carbon or the carbon stored in an organic materials such as wood or plastic	Mass CO_2_/mass material $$ \left(\raisebox{1ex}{$\mathrm{kg}\ {\mathrm{CO}}_2$}\!\left/ \!\raisebox{-1ex}{$\mathrm{kg}$}\right.\right) $$
EC(*t*)_bio_	Time-dependent embodied biogenic carbon of a building material	Mass CO_2_/mass material $$ \left(\raisebox{1ex}{$\mathrm{kg}\ {\mathrm{CO}}_2$}\!\left/ \!\raisebox{-1ex}{$\mathrm{kg}$}\right.\right) $$
[CO_2_]	Carbon dioxide concentration	Mass/vol $$ \left(\raisebox{1ex}{$\mathrm{kg}$}\!\left/ \!\raisebox{-1ex}{$\mathrm{L}$}\right.\right) $$
[CO_2_]	Carbon dioxide concentration as a function of time	Mass/vol $$ \left(\raisebox{1ex}{$\mathrm{kg}$}\!\left/ \!\raisebox{-1ex}{$\mathrm{L}$}\right.\right) $$
[CH_4_]	Methane concentration	Mass/vol $$ \left(\raisebox{1ex}{$\mathrm{kg}$}\!\left/ \!\raisebox{-1ex}{$\mathrm{L}$}\right.\right) $$
[CH_4_](*t*)	Methane concentration as a function of time	Mass/vol $$ \left(\raisebox{1ex}{$\mathrm{kg}$}\!\left/ \!\raisebox{-1ex}{$\mathrm{L}$}\right.\right) $$
[LFG]	Landfill gas concentration [LFG] = [CO_2_] + [CH_4_]	Mass/vol $$ \left(\raisebox{1ex}{$\mathrm{kg}$}\!\left/ \!\raisebox{-1ex}{$\mathrm{L}$}\right.\right) $$
*M*_0_	Mass of biodegradable C&D disposed of in landfills	Mass (kg)
*L*_0_	Methane generating potential of biodegradable in landfills	Vol/mass $$ \left(\raisebox{1ex}{${\mathrm{m}}^3$}\!\left/ \!\raisebox{-1ex}{$\mathrm{kg}$}\right.\right) $$
*k*	Decay rate of biodegradable materials in landfills	1/time $$ \left(\raisebox{1ex}{$1$}\!\left/ \!\raisebox{-1ex}{$\mathrm{year}$}\right.\right) $$
*p*	LFG pressure in landfill	Pressure (atm)
mw_CO2_	Molecular weight of carbon dioxide	Mass/mol $$ \left(\raisebox{1ex}{$\mathrm{g}$}\!\left/ \!\raisebox{-1ex}{$\mathrm{mol}$}\right.\right) $$
mw_CH4_	Molecular weight of methane	Mass/mol $$ \left(\raisebox{1ex}{$\mathrm{g}$}\!\left/ \!\raisebox{-1ex}{$\mathrm{mol}$}\right.\right) $$
*R*	Ideal gas law constant	Vol·pressure/mol·temp $$ \left(\raisebox{1ex}{$\mathrm{L}\bullet \mathrm{atm}$}\!\left/ \!\raisebox{-1ex}{$\mathrm{mol}\bullet \mathrm{K}$}\right.\right) $$
*T*	Temperature inside a landfill	Temp (K)
*P*_CH4_	Percentage of methane in landfill gas	Percent (%)
*Q*_CH4_	Flow rate of methane out of the atmosphere	Vol/time $$ \left(\raisebox{1ex}{$\mathrm{L}$}\!\left/ \!\raisebox{-1ex}{$\mathrm{year}$}\right.\right) $$
*B*	$B=\left[\frac{M_{\mathrm{o}}{L}_{\mathrm{o}}k\bullet p\bullet {\mathrm{mw}}_x}{RT}\right]\kern1em x={\mathrm{CO}}_2\ \mathrm{or}\ {\mathrm{CH}}_4$	

Dividing Eq. 1 by the volume of Earth’s atmosphere (*V*_atm_) gives a differential equation for the concentration of *G* in terms of the GHG residence time (*θ*) in the atmosphere:

2 $$ \frac{dG}{dt}=-\frac{1}{\theta }G $$

The solution for *G*(*t*) from Eq. 2 can be given by:

3 $$ G(t)={G}_0{e}^{-t/\theta } $$

with the initial condition *G*(0) = *G*_0_ at *t* = 0,

where, *G*_0_ is the initial concentration of GHG pulse emitted.

### Modeling pulse GHG emissions

Equation 3 represents the concentration of the GHG in the atmosphere, emitted as pulse during manufacturing or incineration or as a fugitive emission, and subsequently removed from the atmosphere over time. If the source of the GHG pulse is combustion of the material during incineration, then *G*_0_ is given by the mass of the material combusted (*m*) multiplied by the carbon intensity coefficient, which is termed EC_bio_ in the Inventory of Carbon and Energy (ICE) tables compiled by Hammond et al*.* (Hammond 2011). Hammond et al. use the subscript “bio” in the EC_bio_ coefficient to refer to the biogenic carbon stored in wood that is released as CO_2_ upon combustion. In this work, the same coefficient is used to represent the CO_2_ released from any incinerated, flammable organic material, (e.g., plastic and rubber) and not just wood. Equation 4 gives the concentration of GHG emissions associated with the incineration of a mass (*m*) of flammable organic material.

4 $$ {G}_{\mathrm{bio}}(t)=\mathrm{m}\bullet {\mathrm{EC}}_{\mathrm{bio}}{e}^{-t/\theta } $$

### Landfill gas production function

Landfill gas (LFG) production has been previously described by Mohan et al. and EPA (Mohan and MacDonald 2016a b; De La Cruz and Barlaz 2010). LFG production is a complex biological phenomenon carried out by microorganisms. The rate of production and amount of LFG produced by microbes depends on the methane generating potential (*L*_o_) of the material and the microbial decay rate (*k*) of the material. Methane and CO_2_ production rates from decomposing building materials were modeled by a modified solution to a first-order landfill decay equation and are given by Eq. 5 (De La Cruz and Barlaz 2010). In Eq. 5, the physical constants are combined together and collectively termed *B*.

5 $$ \left[{\mathrm{CH}}_4\right](t)=\left[\frac{M_{\mathrm{o}}{L}_{\mathrm{o}}k\bullet p\bullet \mathrm{mw}}{V_{\mathrm{atm}} RT}\right]{e}^{- kt}=\frac{B}{V_{\mathrm{atm}}}{e}^{- kt} $$

$$ \mathrm{where}\ B=\left[\frac{M_{\mathrm{o}}{L}_{\mathrm{o}}k\bullet p\bullet \mathrm{mw}}{RT}\right] $$

The factors contained in *B* are the physical constants that affect the rate of CH_4_ production in a landfill. The constant *M*_0_ is the mass of biodegradable material disposed of in a landfill. The factors mw and *R* represent the molecular weight of CH_4_ (16 g/mol) and the ideal gas law constant (0.0821 L atm/mol K) respectively. The factors *L*_o_ and *k* represent the methane production potential of a particular material and the decay rate of biodegradable material. These constants can only be determined empirically, from both laboratory methods and field surveys, and vary widely (Bogner and Spokas 1993; Hansen et al. 2004; Schirmer et al. 2014). This work utilizes the constants *L*_o_ = 170 m^3^/MT and *k* = 0.05 1/year which are used as parameters for a conventional landfill, in the EPA’s Landfill Gas Emissions Model (LandGEM). Factors *p* and *T* represent the barometric pressure of CH_4_ within the interstices and the temperature within the landfill. For this work, conservative values of *p* = 1 atm and *T* = 20 °C (293 K) are used. Pressure and temperature change with landfill depth and season, but range around 1 atm and 20 °C (Yesiller and Hanson 2003).

Multiplying both sides of Eq. 5 by *V*_atm_ provides the mass of CH_4_ (instead of concentration [CH_4_]) at time *t*, emitted from landfilled material:

6 $$ {\mathrm{CH}}_4(t)=\left[\frac{M_{\mathrm{o}}{L}_{\mathrm{o}}k\bullet p\bullet \mathrm{mw}}{RT}\right]{e}^{- kt}=B{e}^{- kt} $$

Note that the symbols with square brackets represent concentration in mass/volume, while symbols with no brackets represent mass of CH_4_.

Landfill gas is generally considered to be a mixture of 50% CH_4_ and 50% CO_2_. The mass of CO_2_ produced by landfills is derived as a proportion of the mass of CH_4_ produced (EPA 2005; De La Cruz and Barlaz 2010). The amount of CO_2_ in LFG is given as a percentage of CH_4_ (*P*_CH4_) as shown below:

7 $$ \left(1-{P}_{\mathrm{CH}4}\right)\bullet \mathrm{LFG}(t)=\left(1-{P}_{\mathrm{CH}4}\right)\bullet \left[{\mathrm{CH}}_4(t)+{\mathrm{CO}}_2(t)\right]={\mathrm{CO}}_2(t) $$

Rearranging Eq. 7 for CO_2_(*t*) gives an equation for the mass of CO_2_ produced as a proportion of the mass of CH_4_ generated:

8 $$ {\mathrm{CO}}_2(t)=\left[\frac{1}{P_{\mathrm{CH}4}}-1\right]{\mathrm{CH}}_4(t) $$

The LFG(*t*) produced by the landfill is the sum of the masses of CH_4_(*t*) and CO_2_(*t*) as below. To report the GWP (in CO_2_ equivalents) of LFG(*t*) from landfilled materials, CH_4_ is multiplied by 25 to account for its 25-fold increased heat trapping efficiency of methane.

9 $$ \mathrm{LFG}(t)={\mathrm{CO}}_2(t)+25{\mathrm{CH}}_4(t) $$

Substituting Eqs. 7 and 8 into Eq. 9 gives Eq. 10, which provides an expression for GWP (in CO_2_ equivalents) from LFG production in terms of CH_4_ and time.

10 $$ \mathrm{LFG}(t)=\left[\frac{1}{P_{\mathrm{CH}4}}-1\right]{\mathrm{CH}}_4(t)+25{\mathrm{CH}}_4(t)=\left(\frac{1}{P_{\mathrm{CH}4}}+24\right){\mathrm{CH}}_4(t) $$

Substituting Eq. 6, for the mass of CH_4_ produced by a mass of landfilled material (CH_4_(*t*)), in Eq. 10, provides the expression for the time-dependent production of LFG (Eq. 11), based on the mass of the landfilled material and physical constants.

11 $$ \mathrm{LFG}(t)=\left(\frac{1}{P_{\mathrm{CH}4}}+24\right)B{e}^{- kt} $$

### Removal of GHG emissions associated with landfilled building materials over time

When considering the time-dependent removal of GHGs from the material disposed of in a landfill, Eq. 1 (for a pulse input of GHG into the atmosphere) must be modified to include the time-dependent input of LFG into the CSTR as well as the time-dependent removal of the GHGs by Earth’s GHG removal mechanisms (sinks). Methane and CO_2_ emissions are modeled separately and added together at the end, since each gas has a different atmospheric residence time. Equation 12 represents the mass flow of CH_4_ produced by a mass of material in a landfill (*Be*^−*kt*^) flowing into the atmosphere and removal of CH_4_ by natural mechanisms (−*Q*_CH4_[CH_4_]).

12 $$ {V}_{\mathrm{atm}}\frac{d\left[{\mathrm{CH}}_4\right]}{dt}=B{e}^{- kt}-{Q}_{\mathrm{CH}4}\left[{\mathrm{CH}}_4\right] $$

Equation 12 gives the production rate of CH_4_ from anaerobically digested biodegradable organic material. Dividing through by *V*_atm_ and rearranging Eq. 12 gives the non-homogeneous differential equation:

13 $$ \frac{d\left[{\mathrm{CH}}_4\right]}{dt}+\frac{1}{\theta_{\mathrm{CH}4}}\left[{\mathrm{CH}}_4\right]=\frac{B}{V_{\mathrm{atm}}}{e}^{- kt} $$

where *θ*_CH4_ is the atmospheric residence time of CH_4_.

With the initial condition [CH_4_] = 0, at *t* = 0, the solution to Eq. 13 is an explicit, time-dependent expression for the atmospheric concentration of CH_4_.

14 $$ \left[{\mathrm{CH}}_4\right](t)=\frac{B}{V_{\mathrm{atm}}}\left[\frac{1}{\frac{1}{\theta_{\mathrm{CH}4}}-k}\right]\left[{e}^{- kt}-{e}^{-\frac{t}{\theta_{\mathrm{CH}4}}}\right] $$

Multiplying through by *V*_atm_ gives Eq. 15, which is the mass of atmospheric CH_4_ at each moment in time.

15 $$ {\mathrm{CH}}_4(t)=B\left[\frac{1}{\frac{1}{\theta_{\mathrm{CH}4}}-k}\right]\left[{e}^{- kt}-{e}^{-\frac{t}{\theta_{\mathrm{CH}4}}}\right] $$

To model CO_2_ production from landfilled materials, Eq. 8 gives the yield of CO_2_ as a percentage of CH_4_ produced in a landfill, and Eq. 16 gives the CO_2_ mass input from a landfill ($\left[\frac{1}{P_{\mathrm{CH}4}}-1\right]B{e}^{- kt}$), into the atmosphere with an exit (−*Q*_CO2_[CO_2_]) representing Earth’s CO_2_ removal mechanisms.

16 $$ {V}_{\mathrm{atm}}\frac{d\left[{\mathrm{CO}}_2\right]}{dt}=\left[\frac{1}{P_{\mathrm{CH}4}}-1\right]B{e}^{- kt}-{Q}_{\mathrm{CO}2}\left[{\mathrm{CO}}_2\right] $$

Dividing by *V*_atm_ and rearranging Equation 16 gives a non-homogeneous differential, where (${\theta}_{\mathrm{CO}2}=\frac{V_{\mathrm{atm}}}{Q}$) is the atmospheric residence time of CO_2_.

17 $$ \frac{d\left[{\mathrm{CO}}_2\right]}{dt}+\frac{1}{\theta_{\mathrm{CO}2}}\left[{\mathrm{CO}}_2\right]=\left[\frac{1}{P_{\mathrm{CH}4}}-1\right]\frac{B}{V_{\mathrm{atm}}}{e}^{-\mathrm{k}t} $$

The solution to Eq. 17, with the initial condition, [CO_2_] = 0, at *t* = 0, is an expression for the atmospheric concentration of CO_2_ from a mass of landfilled material, as a function of time.

18 $$ \left[{\mathrm{CO}}_2\right](t)=\frac{B}{V_{\mathrm{atm}}}\left[\frac{1}{\frac{1}{\theta_{\mathrm{CO}2}}-k}\right]\left[{e}^{- kt}-{e}^{-\frac{t}{\theta_{\mathrm{CO}2}}}\right] $$

Multiplying through by *V*_atm_ provides the mass of CO_2_ produced from the landfilled material.

19 $$ {\mathrm{CO}}_2(t)=B\left[\frac{1}{\frac{1}{\theta_{\mathrm{CO}2}}-k}\right]\left[{e}^{- kt}-{e}^{-\frac{t}{\theta_{\mathrm{CO}2}}}\right] $$

The total time-dependent GWP of the landfill gas emitted by material (in kgCO_2_e) is given by Eq. 9, where the mass of CH_4_ produced is multiplied by 25 to account for methane’s 25-fold greater heat trapping capacity. Substituting Eqs. 15 and 19 into the CH_4_ and CO_2_ masses in Eq. 9 gives Eq. 20, which is the GWP due to CH_4_ and CO_2_ (LFG) produced in time by a mass of landfilled material.

20 $$ \mathrm{LFG}(t)=B\left\{\left[\frac{25}{\frac{1}{\theta_{\mathrm{CH}4}}-k}\right]\left[{e}^{- kt}-{e}^{-\frac{t}{\theta_{\mathrm{CH}4}}}\right]+\left[\frac{1}{P_{\mathrm{CH}4}}-1\right]\left[\frac{1}{\frac{1}{\theta_{\mathrm{CO}2}}-k}\right]\left[{e}^{- kt}-{e}^{-\frac{t}{\theta_{\mathrm{CO}2}}}\right]\right\} $$

### Lifetime embodied carbon GHG emissions associated with building materials

The GWP for the lifetime of a building material is the sum of the GHG emitted during manufacture (Eq. 3) and the end-of-life treatment: (i) incineration (Eq. 4) or (ii) landfill disposal (Eq. 20). The time of demolition (*T*_Demo_) of a building is the time span from its construction to its demolition. The amount of atmospheric GHG, due to the manufacture, demolition at *T*_Demo_, with incineration disposal of the material is given by Eq. 21.

21 $$ {G}_{\mathrm{I}}(t)=G(t)+{G}_{\mathrm{bio}}(t)={G}_0{e}^{-\frac{t}{\theta_{\mathrm{CO}2}}}+{G}_{\mathrm{bio}}(t)\left\{\begin{array}{c}0\kern9.25em \mathrm{for}\ t<{T}_{\mathrm{Demo}}\\ {}m\bullet {\mathrm{EC}}_{\mathrm{bio}}{e}^{-t/{\theta}_{\mathrm{CO}2}}\kern0.5em \mathrm{for}\ t\ge {T}_{\mathrm{Demo}}\end{array}\right. $$

Equation 22 represents the amount of atmospheric GHG remaining from the manufacture and landfill disposal of C&D debris at *T*_Demo_ (*G*_*L*_(*t*) ).

22 $$ {G}_L(t)=G(t)+\mathrm{LFG}(t)={G}_0(t){e}^{-\frac{t}{\theta_{\mathrm{CO}2}}}+\mathrm{LFG}(t)\left\{\begin{array}{c}0\kern3.25em \mathrm{for}\ t<{T}_{\mathrm{Demo}}\\ {}\mathrm{LFG}(t)\kern0.5em \mathrm{for}\ t\ge {T}_{\mathrm{Demo}}\end{array}\right. $$

### Embodied carbon GHG emissions associated with entire building, over time

Equation 23 provides the time-dependent (*G*(*t*)_build_), the sum of the *G*(*t*) values for manufacture of each building component plus the end-of-life treatments (incineration or landfill:

23 $$ {G}_{\mathrm{build}}(t)=\sum G(t)+\left\{\begin{array}{c}0\kern3.75em \mathrm{for}\ t<{T}_{\mathrm{Demo}}\\ {}\sum {G}_I(t)\kern1em \mathrm{for}\ t\ge {T}_{\mathrm{Demo}}\\ {}\sum {G}_L(t)\kern1em \mathrm{for}\ t\ge {T}_{\mathrm{Demo}}\end{array}\right. $$

where, *G*(*t*) = GHG emissions from manufacture of all materials in the building.

*G*_I_(*t*) = GHG emissions from combustion of incinerable materials.

*G*_*L*_(*t*) = GHG emissions from landfill disposal.

### Global warming impact due to materials

In this work, a new term, Global Warming Impact of Materials (GWIM), is introduced and defined as below:

“Global Warming Impact of Materials (GWIM) includes all GHG emissions associated with a material including its manufacture (EC), and end-of-life treatment of demolition debris, over a time until they are reduced to zero.”

The GWIM of a material or an assembly of materials is determined from the area under the GHG emissions vs time profiles for materials or assemblies of materials (Eqs. 21, 22, and 23) and is given as Eq. 24.

24 $$ \mathrm{GWIM}={\int}_0^{T_{\infty }}G(t) dt $$

In the CSTR model, the atmospheric residence time of molecules is a statistical distribution of the lifetime of molecules in the reactor. The mean of this distribution is the residence time for each GHG (Trussell and Hand 2005). Because this is a statistical distribution, some of the GHG molecules will remain forever in the reactor. The mathematical exponential lifetime GHG emission models show that at 700 years, there are less than 1% of the original GHG molecules left in the atmosphere. Therefore, for purposes of calculating GWIM values, it is assumed that *T*_∞_ = 700 years.

### Productive and non-productive impact of GHG emissions associated with materials

Any material that is manufactured has an impact on the Earth’s environment. Even the most environmentally friendly materials and manufacturing processes consume raw materials and require energy to produce. In this research, “productive GWIM” (GWIM_p_) is defined as the GWIM of materials during the time that the material or building is being utilized. Conversely, a non-productive GWIM (GWIM_np_) is defined as the GWIM after a building is demolished at *T*_Demo_, at the end of its service life. After a building is demolished, some of the embodied carbon from manufacturing of the materials remains in the atmosphere. The remaining emissions can be considered a squandering of the original GHG emissions associated with the materials. These emissions provide no positive value and only negatively affect society by causing global warming. Greenhouse gas emissions from incineration or landfilling of debris are all considered non-productive as they only enhance global warming, but provide no use of the facility. It should be clarified that productive and non-productive do not imply any goodness or badness to the building, instead productive simply implies the material or building is being used and non-productive means it is not. Equations 25 and 26 give the productive and non-productive GWIMs:

25 $$ {\mathrm{GWIM}}_{\mathrm{p}}={\int}_0^{{\mathrm{T}}_{\mathrm{Demo}}}G(t) dt $$

26 $$ {\mathrm{GWIM}}_{\mathrm{np}}={\int}_{T_{\mathrm{Demo}}}^{T_{\infty }}G(t) dt $$

Added together, GWIM_p_ and GWIM_np_ give the total GWIM for a material or assembly of materials (Eq. 24). The GWIM_p_ and GWIM_np_ are of particular importance when describing materials such as cement or glass that have outstanding emissions from their manufacture, but do not emit GHGs after they are disposed of in landfills, against wood or plastics that emit GHGs after they are demolished and either incinerated or landfilled.

### Simulations of lifetime GHG emissions for various service lives and various end-of-life treatments for a residential building

The simulations were performed using Microsoft® EXCEL spreadsheet. Integration of the GWIM curves was performed using the trapezoidal rule with the time step set to 0.25 years. In this paper, the lumber portion of the building materials was modeled for demonstrating the simulations. Modeling and computing lifetime GHG emissions, two service lives were selected: 50 years and 100 years, and three most used end-of-life treatments were selected for computations and for making objective comparisons between the treatments: incineration, landfill disposal, and deconstruction.

## Simulation results

### Lifetime greenhouse gas emissions resulting from manufacturing (EC), service lives, and end-of-life treatments

#### The structure of the example building used

The example structure, in this paper, is modeled on a residential house in Buffalo, NY. The house is a balloon-framed, wooden structure built about 1900 AD. The plan of the building is a 22 ft × 50 ft (6.7 × 15.2 m) rectangle, 2 stories tall with a 12:12 pitch roof. The house retains most of its original wood siding and moldings. The structure is composed of old-growth Eastern Hemlock (*Tsuga canadensis*) that was cut to true dimensions, e.g., a 2 × 4 stud (5.08 cm × 10.2 cm) is 2 in. (5.08 cm) thick by 4 in. (10.2 cm) wide unlike modern lumber where a 2 × 4 stud is 1½ (3.8 cm) in. thick by 3½ (8.9 cm) wide. The house has 14 interior doors and 3 exterior doors made of Southern yellow or longleaf pine (*Pinus palustris*). It has 14, wooden-sash framed exterior windows, made from Eastern white pine (*Pinus strobus*), jack pine (*Pinus banksiana*), loblolly pine (*Pinus taeda*), or similar wood. The foundation is 8 ft (2.4 m) high and composed of large, rough cut blocks of Onondaga limestone. Their lengths and widths vary but their thickness averages 1 ft (0.304 m). The basement floor is 5 in. (12.7 cm) poured cement concrete, thought to have been added in the 1920s. The flooring is ¾ in. (1.09 cm) thick by 2 in. (5.08 cm) wide strips, red oak over a subfloor of 1 in. (2.54 cm) thick by 6 in. (15.2 cm) wide pine (Eastern, jack, loblolly, or other) or hemlock planking. The baseboard, window, and door trim are made from Southern yellow pine. The interior walls of the house are composed of the original sand-cement plaster which averages ½ in. (1.27 cm) thick and is attached to ½ in. (1.27 cm) thick by 2 in. (5.08 cm) wide hemlock lath strips. The exterior siding is original, 6 in. (15.2 cm) wide pine or cedar clapboard. Finally, there are two chimneys made of common, cut, red clay brick, 2 in. (5.08 cm) thick, 4 in. (10.2 cm) wide by 8 in. (20.4 cm) long. The front chimney, built into a fireplace, is irregularly shaped and is more massive than the rear chimney which is 1½ ft (0.457 m) by 1½ ft (0.457 m) square. Both chimneys start in the basement and rise 3 ft (0.91 m) above the ridge line of the roof. In this work, the GWIM values for the lumber component of the house were determined for two service lives of 50 and 100 years, i.e., *T*_Demo_ denotes the time of demolition. Additionally, the effect of three end-of-life treatments was included: incineration, landfill disposal, and deconstruction. Deconstruction was considered as an alternative to conventional demolition and disposal. The lumber left after deconstruction and recovery was either incinerated or disposed of in a landfill.

#### Example material—lumber

The example building contains approximately 45,620 boardfeet (BF) (107.7 m^3^) of softwood lumber and 3300 BF (7.79 m^3^) of hardwood lumber (total 48,920 BF [115.5 m^3^]). Pine and hemlock softwoods have a density range of 25–35 lb/ft^3^ (400 to 550 kg/m^3^) and an average density of 30 lbs/ft^3^ (475 kg/m^3^). Red oak has an average density of about 45 lbs/ft^3^ (720 kg/m^3^). Using these densities, the masses of the softwood and hardwood lumber, in the example building are 81,723 kg and 5613 kg respectively. The embodied carbon (EC) and EC_bio_ values used in this research were taken from the Inventory of Carbon and Energy (ICE) v 2.0 tables published by Hammond et al. (Hammond 2011). Using these tables, sawn softwoods have an EC value of 0.2 kgCO_2_e/kg Hardwood has an EC value of 0.24 kgCO_2_e/kg and EC_bio_ values of 0.63. The atmospheric lifetime of CO_2_ is set to 125 years, which is the mean of the range 50–200 years reported by the EPA (EPA 2017). The lifetime GHG emissions have been computed for the 48,920 BF of lumber, in this paper, using mathematical models presented in the earlier sections.

### Plotting lifetime GHG emissions associated with lumber

#### Lifetime GHG emissions from embodied carbon for service lives of 50 and 100 years

**Fig. 2:**
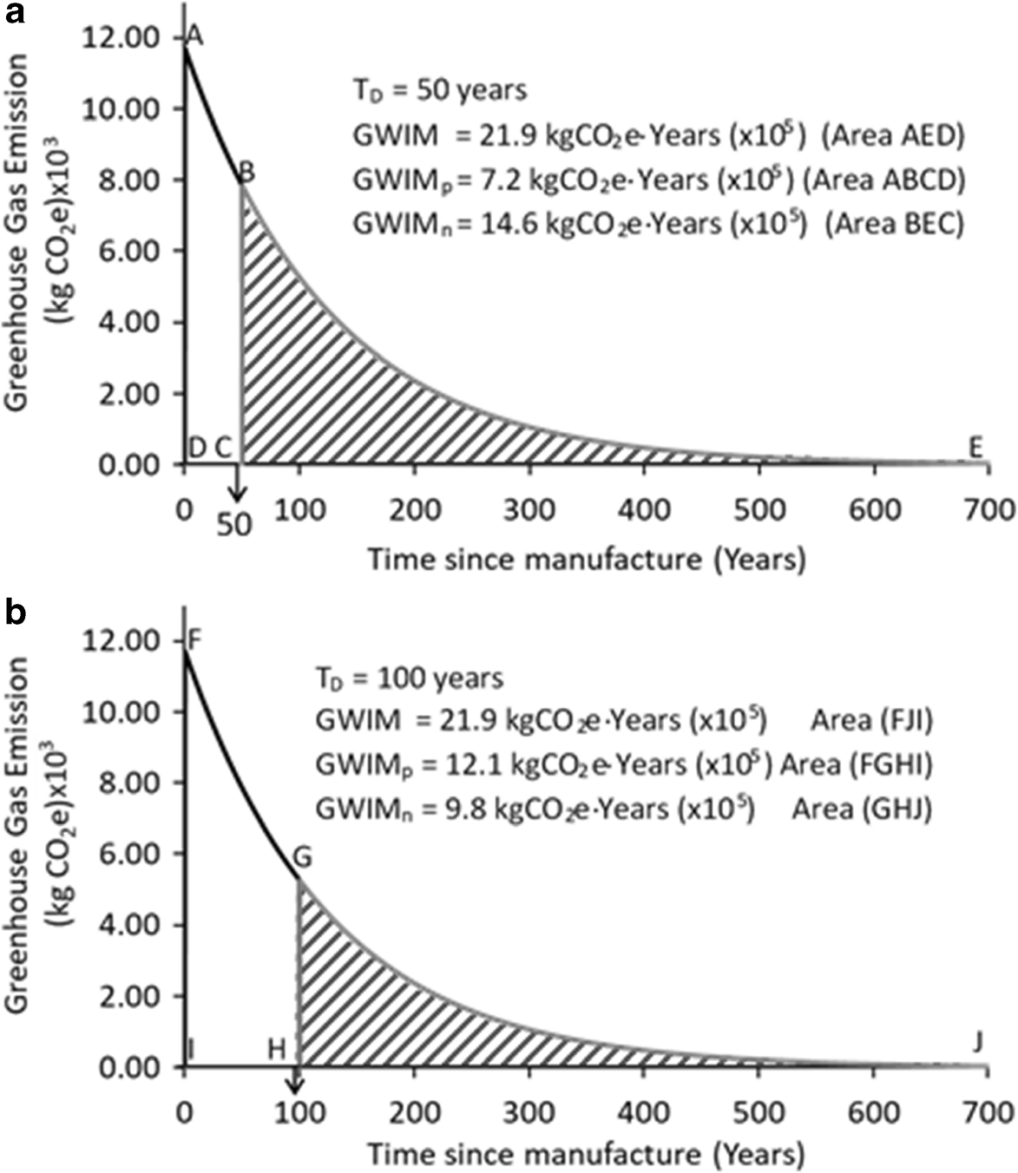
a Lifetime GHG emissions associated with building materials for embodied carbon (EC) (*T*_Demo_ = 50 years). **b** Lifetime GHG emissions associated with building materials for embodied carbon (EC) (*T*_Demo_ = 100 years)

The GHG emissions, over time, associated with the manufacture of lumber from the example building are shown in Fig. 2a for *T*_Demo_ = 50 years and in Fig. 2b for *T*_Demo_ = 100 years. If demolition was not performed (the “do nothing” scenario) and the material was utilized indefinitely, the GHG emissions remaining in the atmosphere will decrease over time, as shown by the exponential lines in Fig. 2a, b.

The exponential GHG decay curves, line ABE in Fig. 2a and line FGJ in Fig. 2b, are based on decay rates calculated by Eq. 3. For the lumber in the example structure, the total GWIM is calculated to be 21.9 × 10^5^ kgCO_2_e-years $\left[\mathrm{GWIM}={\int}_0^{\infty \mathrm{years}}{G}_0{e}^{-t/\left(125\ \mathrm{years}\right)} dt\right]$ where *G*_o_ is EC coefficients for softwood (0.2 kgCO_2_e/kg) and for hardwood (0.24 kgCO_2_e/kg) multiplied by the masses of the softwood (81,723 kg) and hardwood (5613 kg), respectively, in the example structure.

In Fig. 2a, the unshaded region (ABCD) represents the productive portion of GWIM or GWIM_p_, and the shaded region (area BEC) represents the non-productive portion of GWIM or GWIM_np_ of 48,920 BF of lumber from the example house, if *T*_Demo_ = 50 years. The GWIM in this figure includes only GHG emissions from the manufacture (EC) and GHG reduction during the service life of the material. Emissions from end-of-life treatments are not included in Fig. 2a, b. The GWIM values are computed as below.

For *T*_Demo_ = 50 years:

GWIM = 21.9 × 10^5^ kgCO_2_e-years

GWIM_p_ = 7.2 × 10^5^ kgCO_2_e-years, and

GWIM_np_ = 14.6 × 10^5^ kgCO_2_e-years.

In Fig. 2b, the unshaded region (area FGHI) represents the GWIMp and the shaded region (area GJH) represents the GWIM_np_ of 48,920 BF of lumber for *T*_Demo_ = 100 years. The GWIM values are computed as below:

For *T*_Demo_ = 100 years:

GWIM = 21.9 × 10^5^ kgCO_2_e-years

GWIM_p_ = 12.1 × 10^5^ kgCO_2_e-years, and

GWIM_np_ = 9.8 × 10^5^ kgCO_2_e-years.

Extending the service life of the structure from 50 to 100 years, increased the productive GWIM (GWIM_P_) by 68%, from 7.2 × 10^5^ to 12.1 × 10^5^ kgCO_2_e-years, and GWIMnp reduced 33% from 14.6 × 10^5^ to 9.8 × 10^5^ kgCO_2_e-years.

#### Lifetime GHG emissions from embodied carbon and incineration as end-of life treatment for service lives of 50 and 100 years

**Fig. 3:**
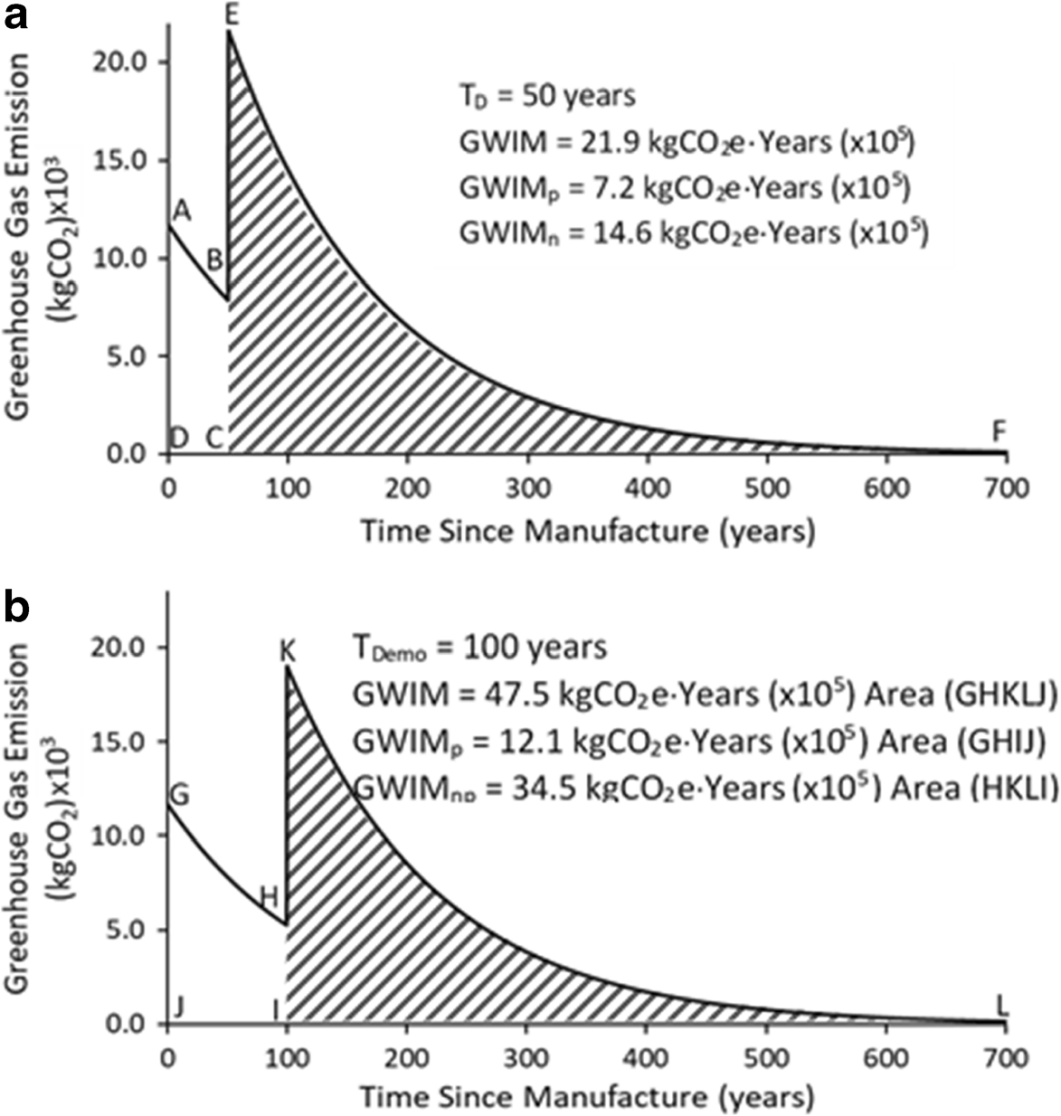
**a** Lifetime GHG emissions associated with building materials for EC and incineration as end-of-life treatment of demolition debris (*T*_Demo_ = 50) years). **b** Lifetime GHG emissions associated with building materials for EC and incineration as end-of-life treatment of demolition debris (*T*_Demo_ = 100 years)

**Fig. 4:**
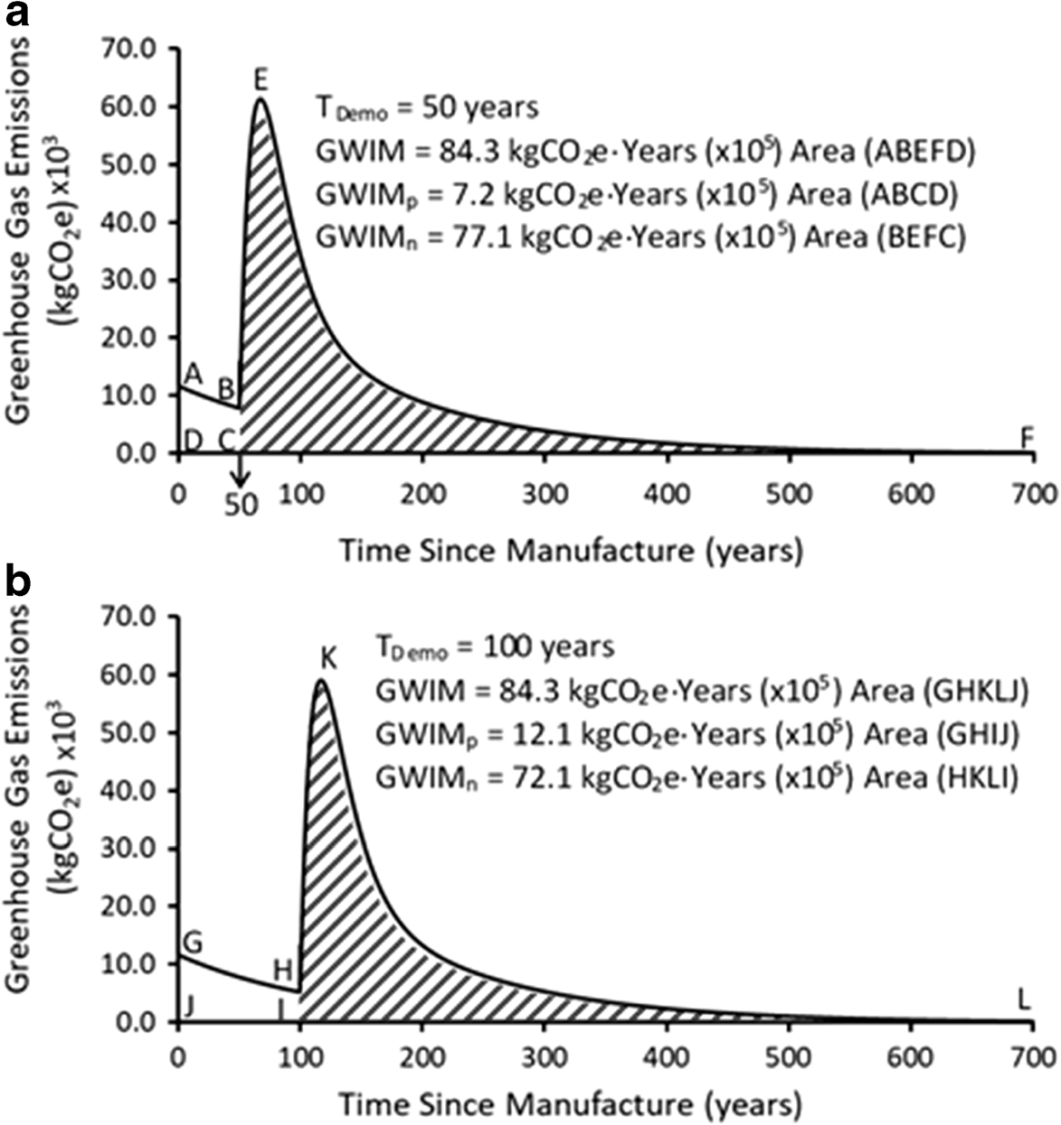
**a** Lifetime GHG emissions associated with building materials for EC and landfill disposal as end-of-life treatment of demolition debris (*T*_Demo_ = 50 years). **b** Lifetime GHG emissions associated with building materials for EC and landfill disposal as end-of-life treatment of demolition debris (*T*_Demo_ = 100 years) years

**Fig. 5:**
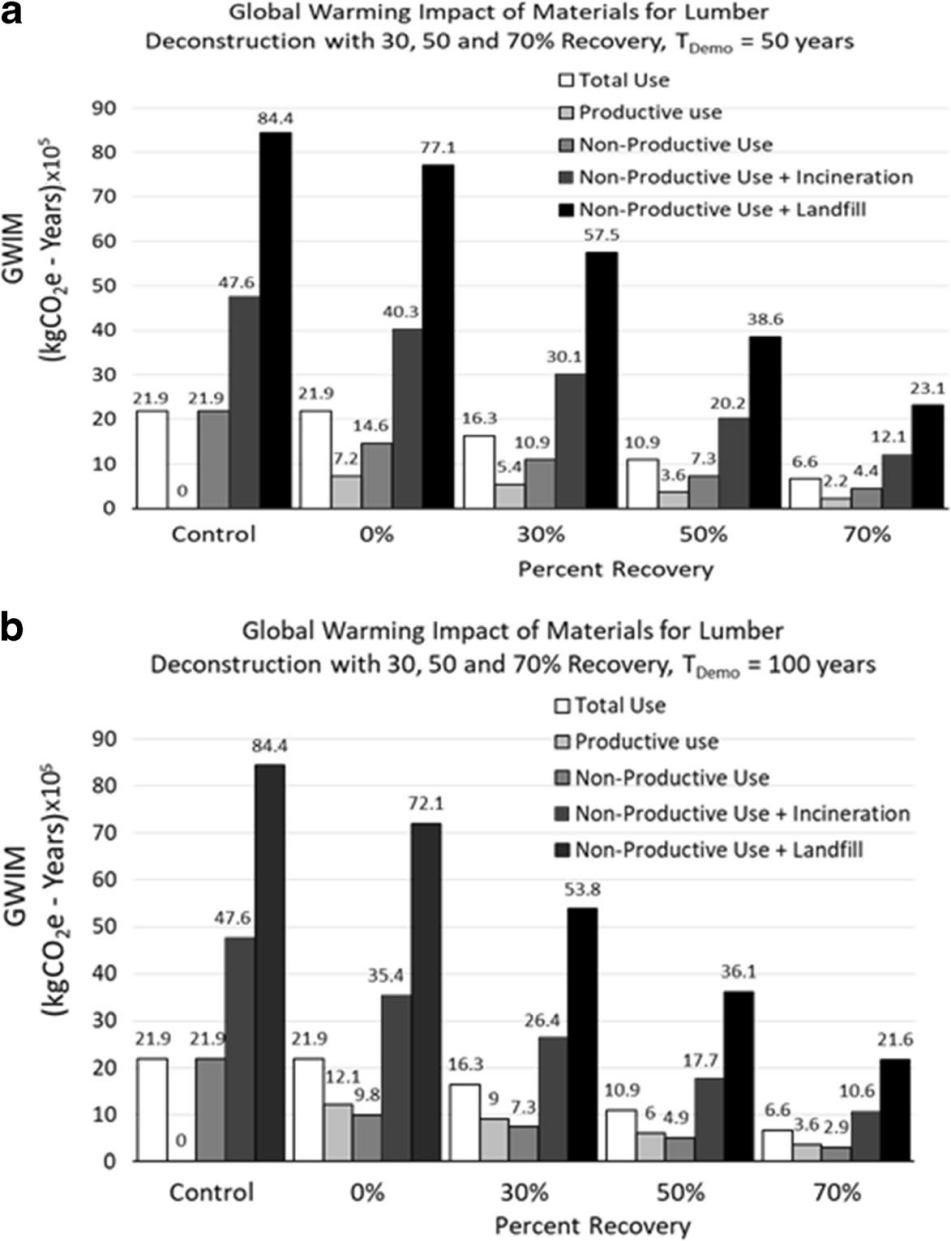
**a** The Global Warming Impact of Materials (GWIM) for 48,920 BF of lumber from the example house. Deconstruction with 30%, 50%, and 70% recovery (*T*_Demo_ = 50 years). **b** The Global Warming Impact of Materials (GWIM) for 48,920 BF of lumber from the example house. Deconstruction with 30%, 50%, and 70% recovery (*T*_Demo_ = 100 years)

Figures 3, 4, and 5 show the effect of the EC, the service lives of the material, and the end-of-life treatment of the demolition debris after the building becomes obsolete and is demolished. The three possible end-of-life treatments, (i) incineration, (ii) landfill disposal, or (iii) deconstruction, have been considered.

In Fig. 3a, the building is demolished at 50 years (*T*_Demo_ = 50) and all of the construction and demolition (C&D) debris is incinerated. The steep, vertical steps in Fig. 3a, b represent moment of incineration, which is an infinitely short process compared with the atmospheric lifetime of the GHGs. The GWIM values, in Fig. 3a, are calculated from Eq. 21. The total GWIM for this structure, at *T*_Demo_ = 50 years and incineration as the end-of-life treatment, is 66.2 × 10^5^ kgCO_2_e-years. The unshaded region (area ABCD), 7.2 × 10^5^ kgCO_2_e-years represents the productive portion, GWIM_p_ for the material, and the shaded region (area EFC), 58.9 × 10^5^ kgCO_2_e-years, represents the GWIM_np_ of the lumber from the example house. If the houses were aged to 100 years at the time of demolition and the lumber were incinerated as the end-of-life treatment, the total GWIM is 66.0 × 10^5^ kgCO_2_e-years. The unshaded region in Fig. 3b (area GHIJ), 12.1 × 10^5^ kgCO_2_e-years, represents the GWIM_p_, and the shaded region (area KLI), 54.0 × 10^5^ kgCO_2_e-years, represents the GWIM_np_. The GWIM values are summarized below:

For *T*_Demo_ = 50 years:

GWIM = 66.2 × 10^5^ kgCO_2_e-years

GWIM_p_ = 7.2 × 10^5^ kgCO_2_e-years, and

GWIM_np_ = 58.9 × 10^5^ kgCO_2_e-years.

For *T*_Demo_ = 100 years:

GWIM = 66.0 × 10^5^ kgCO_2_e-years

GWIM_p_ = 12.1 × 10^5^ kgCO_2_e-years, and

GWIM_np_ = 54.0 × 10^5^ kgCO_2_e-years.

#### Lifetime GHG emissions from embodied carbon and landfill as end-of life treatment for service lives of 50 and 100 years

Figure 4a, b represents the lifetime GHG emissions of the lumber from the example house, if the demolition debris is disposed of in landfills, at the end of the building’s service life. Two service lives, *T*_Demo_ = 50 years and *T*_Demo_ = 100 years, are used in this demonstration. In Fig. 4a, the GHG emissions associated with manufacture of the lumber and landfill disposal at *T*_Demo_ = 50 years are shown. The exponential decay curve and GWIM values were calculated using Eq. 22. The total GWIM for this structure with *T*_Demo_ = 50 years and landfill disposal as the end-of-life treatment is 84.3 × 10^5^ kgCO_2_e-years. The unshaded region (area ABCD) represents the GWIM_p_ (7.2 × 10^5^ kgCO_2_e-years) whereas the shaded region (area BEFC) represents the GWIM_np_ (77.1 × 10^5^ kgCO_2_e-years). If the house is aged out to 100 years, the GWIM, GWIM_p_, and GWIM_np_ values are 84.2. 12.1, and 72.1 × 10^5^ kgCO_2_e-years respectively. The results are summarizes below.

For *T*_Demo_ = 50 years:

GWIM = 84.3 × 10^5^ kgCO_2_e-years

GWIM_p_ = 7.2 × 10^5^ kgCO_2_e-years, and

GWIM_np_ = 77.1 × 10^5^ kgCO_2_e-years.

For *T*_Demo_ = 100 years:

GWIM = 84.3 × 10^5^ kgCO_2_e-years

GWIM_p_ = 12.1 × 10^5^ kgCO_2_e-years, and

GWIM_np_ = 72.1 × 10^5^ kgCO_2_e-years.

#### Deconstruction at the end-of-life demolition

Deconstruction is a process that recovers usable materials at the time of demolition for reuse, recycle, or repurpose. It has been demonstrated that 30% to 70% of the material can be recovered by deconstruction. If the recovered material is reused in new constructions or renovations, the EC of the reused material is used in place of EC for new material, thus lowering the EC of the new structure. The remaining demolition debris, left after deconstruction, is incinerated or landfilled.

Figure 5a presents the GWIM, GWIM_p_, and GWIM_np_ for the service life of 50 years, and Fig. 5b for 100 years. Figure 5a gives the various GWIM values for three recovery rates via deconstruction: 30%, 50%, and 70%. The numbers given above the bars in Fig. 5a, b are tabulated in Table 2 for comparing them to the recovery rate of 0% recovery when no deconstruction is done. Table 2 concludes the following:

(i) For *T*_Demo_ = 50 years, GWIM increases from 21.9 × 10^5^ to 66.2 × 10^5^ kgCO_2_e-years (+ 202%) if the end-of-life debris is incinerated, and the GWIM increases from 21.9 × 10^5^ to 84.3 × 10^5^ kgCO_2_e-years (+ 285%) if landfill disposal is used.
(ii) A 303% increase occurs in the non-productive (GWIM_np_) portion of GWIM when incineration is used for demolition debris, and 428% if the demolition debris is disposed of in landfills.

**Table 2:** Global warming impact of materials in kgCO_2_e-years (×10^5^) for (i) three end-of-life treatments, (ii) service lives (*T*_Demo_) of 50 and 100 years, and (iii) deconstruction with material recovery at 30, 50, and 70%

EC and end-of-life treatments	GWIM		GWIM_p_		GWIM_np_	
	*T*_Demo_ = 50	*T*_Demo_ = 100	*T*_Demo_ = 50	*T*_Demo_ = 100	*T*_Demo_ = 50	*T*_Demo_ = 100
EC only	21.9	21.9	7.2	12.1	14.6	9.8
EC and incineration	66.2 (+ 202%)^b^	66.0 (+ 201%)^b^	7.2	12.1	58.9 (+ 303%)^b^	54.0 (+ 451%)^b^
EC and landfill	84.3 (+ 285%)^b^	84.2 (+ 284%)^b^	7.2	12.1	77.1 (+ 428%)^b^	72.1 (+ 636%)^b^
EC & deconstruction (recovery 30%)						
Add incineration^a^	52.9 (− 20.0%)^c^	52.8 (− 20.0%)^c^	11.6 (+ 61.1%)^c^	15.0 (+ 24.0%)^c^	41.3 (− 29.9%)^c^	37.8 (− 30.0%)^c^
Add landfill^a^	65.6 (− 22.2%)^d^	65.5 (− 22.2%)^d^	11.6 (+ 61.1%)^d^	15.0 (+ 24.0%)^d^	54.0 (− 30%)^d^	50.5 (− 30.0%)^d^
EC & deconstruction (recovery 50%)						
Add incineration^a^	44.0 (− 33.5%)^c^	44.0 (− 33.3%)^c^	14.6 (+ 103%)^c^	17.0 (+ 40.5%)^c^	29.5 (− 49.9%)^c^	27.0 (50.0%)^c^
Add landfill^a^	53.1 (− 37.0%)^d^	53.0 (37.1%)^d^	14.6 (+ 103%)^d^	17.0 (+ 40.5%)^d^	38.5 (− 50.1%)^d^	36.1 (50.0%)^d^
EC & deconstruction (recovery 70%)						
Add incineration^a^	35.2 (− 46.8%)^c^	35.1 (− 46.8%)^c^	17.5 (+ 143%)^c^	18.9 (+ 56.2%)^c^	17.7 (− 69.9%)^c^	16.2 (− 70.0%)^c^
Add landfill^a^	40.6 (− 51.8%)^d^	40.6 (− 51.8%)^d^	17.5 (+ 143%)^d^	18.9 (+ 56.2%)^d^	23.1 (− 70.0%)^d^	21.6 (− 70.0%)^d^

^a^In deconstruction, the material remaining after recovery is either incinerated or landfilled

^b^Percentage represents change from “EC only”

^c^Percentage represents change from “EC and incineration”

^d^Percentage represents change from “EC and landfill”

**Fig. 6:**
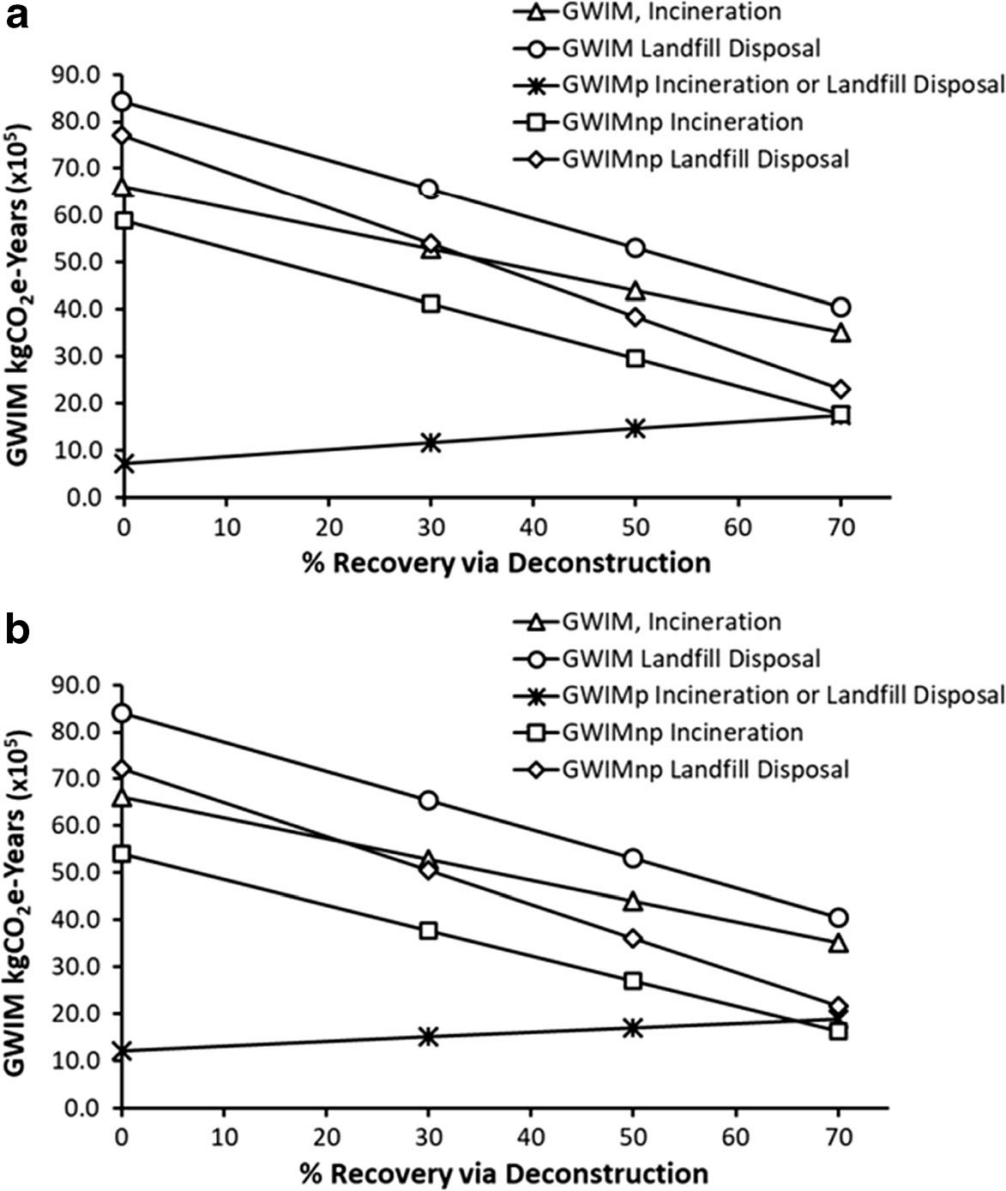
**a** GWIM, GWIM_p_, and GWIM_np_ values as functions of recovery percentages after deconstruction. Lumber was incinerated, disposed of in a landfill, or deconstructed with 30, 50, or 70% recovery of materials (*T*_Demo_ = 50 years). **b** GWIM, GWIM_p_, and GWIM_np_ values as functions of recovery percentages after deconstruction. Lumber was incinerated, disposed of in a landfill or deconstructed with 30, 50, or 70% recovery of materials (*T*_Demo_ = 100 years)

Deconstruction saves on GWIM values as follows:

(i) For 30% recovery, the GWIM is reduced by 20% for incineration and 22.2% if the debris is disposed of in landfills.
(ii) Fifty percent material recovery reduces the GWIM by 33.5% for incineration, and 37% for landfill disposal of the demolition debris. These numbers increase to 46.8% for incineration and 51.8% for landfill disposal, for 70% recovery of materials using deconstruction.
(iii) For the productive portion of GWIM (GWIM_p_), 30% material recovery adds 61.1% GWIM_p_ for incineration and landfill disposal. For 50% recovery, the GWIM_p_ for both treatments increases by 103%, and for 70% recovery, both treatments increase the GWIM_p_ by 143%. In the case of non-productive portion of GWIM (GWIM_np_), 30% material recovery reduces the GWIM_np_ by 30% for both incineration and landfill disposal of demolition debris. Fifty percent recovery reduces the GWIM_np_ by 50% for both end-of-life treatments, and 70% material recovery reduces the GWIM_np_ by 70%. Similar patterns in GWIM, GWIM_p_, and GWIM_np_ follow for *T*_Demo_ = 100 years, for 30%, 50%, and 70% recovery in dconstruction. These numbers are described in Table 2.
(iv) Figure 6a, b displays the reductions in GWIM, GWIM_p_, and GWIM_np_ values for 30%, 50%, and 70% material recovery rates after deconstruction. It can be seen that the GWIM values reduce with increasing material recovery rates, in the cases of incineration or landfill disposal of the remaining demolition debris. Also, the non-productive portion (GWIM_np_) reduced with increasing recovery percentage. However, the productive portion (GWIM_p_) increased with increasing recovery rate. All of the above trends follow the same patterns for both service lives of 50 years and 100 years.

## Summary and conclusions

This research has developed mathematical models for computing lifetime greenhouse gas (GHG) emissions associated with materials. The models include embodied carbon (EC) emissions from the manufacture of materials, and GHG emissions from incineration, or landfill gas (LFG) production from disposal of the material after their service lives. These models are applicable for estimating the global warming impact of any material; however, in this work, they have been used to estimate the global warming impact of lumber from an example residential house. The example structure was modeled with 50- and 100-year service lives to determine the global warming impact of using buildings longer. The global warming impact of the materials, due to their end-of-life treatments (incineration, landfill disposal, or reclamation after deconstruction), was also measured.

This paper introduces a new metric for lifetime GHG emissions associated with materials termed “Global Warming Impact of Materials (GWIM).” The GWIM includes the global warming impact of the manufacturing emissions before the service life and the end-of-life emissions after the service life of the building. The GWIM is subdivided into two portions which represent (i) productive portion (GWIM_p_) that includes the materials’ emissions before the service life of the facility and (ii) non-productive portion (GWIM_np_) which includes the materials’ GHG emissions after the service life until they are reduced to zero. Currently, the global warming impact of materials is given as a static number (kgCO_2_e or MTCO_2_e) based on the EC of a material. The models developed in this report give the GWIM in units of kgCO_2_e-years or MTCO_2_e-years, which includes the time of use of the facility.

Using the models to calculate the GWIMs for an example residential house, this research has determined that increasing the service life of a building from 50 to 100 years (assuming 100% incineration or landfill disposal of the debris) increases the GWIM_p_ 68.1% and decreases the GWIM_np_ 8.32% and 6.5% for incineration and landfill respectively.

The models have also been used to compute the effects of deconstructing the example residential house and recovering 30%, 50%, and 70% of the material during demolition. After a 50-year service life, a 30% recovery (assuming the remaining 70% was incinerated or landfilled) affected a GWIM savings of 20% and 22.2% for diversion from incineration and landfill respectively. If at a service life of 50 years deconstruction was performed with a 50% recovery, these values decrease to 33.5% and 37% compared with no deconstruction (0% recovery). If deconstruction is performed with 70% recovery, the GWIM is reduced by 46.8% and 51.8% for incineration and landfill disposal respectively.

In conlcusion, mathematical models can be developed to accurately measure GHG emissions associated with materials at any point in the life of the material, and for any end-of-life treatment: incineration, landfill, or deconstruction. Applied to several alternative materials, these models can help in the decisions to select carbon-efficient materials at the planning stage of the project. These models can also help in determining decisions to rebuild or renovate a project.

The GHG savings due to deconstruction have not been compared and reported in the literature. Models developed in this research have been computed and plotted GHG savings by deconstruction for material recoveries of 30%, 50%, and 70%. The results show the following:

(i) Thirty percent material recovery saves approximately 20% GHG savings for both service lives of 50- and 100 years for incineration end-of-life treatment and 22% for landfill disposal.
(ii) Fifty percent material recovery saves 34% for both service lives of 50 and 100 years, for incineration end-of-life treatment of the remaining material, and 37% for landfill disposal.
(iii) For 70% material recovery, the GHG savings are 47% for both service lives of 50 and 100 years, for incineration and 52% for landfill disposal of the remaining material after material recovery.

The benefits of reclaiming materials, or deconstruction, are not yet well established. The models can compute GHG savings for different material recovery rates. Also, the cost of deconstruction can be estimated and optimal decisions for deconstruction can be made to save on costs and/or on carbon savings.
